# A Point for Sanatoria Committees

**Published:** 1914-01-24

**Authors:** E. H. Grove

**Affiliations:** Clerk. The Stourbridge and Halesowen Hospital Committee, Halesowen


*To the Editor of* The Hospital.

### A Point for Sanatoria Committees.

*To the Editor of* The Hospital.

Sir,—I am directed by the Committee to state that they take exception to a paragraph which appeared in your issue of December 20 last on page 302 under the startling heading of "'Starved to Death' in a Sanatorium," and the concluding words of which imply that because the Committee passed no resolution they were only half-hearted in their expressed opinion that no blame could be attached to their officials.

The Committee think that your headline might have shown that the "starved to death" was only an allegation, and that the paragraph should have more clearly stated that the complaint of being "starved to death" related to the cold caused by the open-air treatment which was necessary in dealing with cases of tuberculosis.

The Committee further wish me to state that there was no formal complaint. They simply had before them certain rumours which were circulating in a portion of their district, and which were founded on the double meaning of the phrase "starved to death." The whole matter rested on a misapprehension. Under those circumstances there was absolutely no need for them to pass a resolution. They were quite unanimous in their expressions of satisfaction with the staff and the latter's treatment of patients.

The Committee feel sure that, in justice to them and their officials, you will in your paper make quite clear the position, and thus remove from the minds of your readers any idea that they may have that there has been "starvation" at the hospital, or that the Committee are not absolutely satisfied with the treatment of patients by the staff.—Yours faithfully, E. H. Grove, Clerk.

The Stourbridge and Halesowen Hospital Committee,
Halesowen.
January 20, 1914.

[The Committee mistake our point. We were and are of the opinion that when rumours are formally discussed at a meeting, and the Committee concur that they are unfounded, they should be formally repudiated. It was not the half-heartedness of the Committee which we criticised, but the informal method that they adopted to express their satisfaction with the sanatorium staff.—Ed., The Hospital.]

